# The Relationship Between Resilience Level and Perceived Stress Intensity Among Psychiatric Nurses

**DOI:** 10.3390/healthcare13212746

**Published:** 2025-10-29

**Authors:** Kinga Kołodziej, Ewa Wilczek-Rużyczka, Anna Majda

**Affiliations:** 1 Department of Theory and Fundamentals of Nursing, Institute of Nursing and Midwifery, Faculty of Health Sciences, Jagiellonian University Medical College, Michałowskiego 12 St., 31-126 Cracow, Poland; anna.majda@uj.edu.pl; 2 Faculty of Psychology, SWPS University, al. Jana Pawła II 39A, 31-864 Cracow, Poland; ewilczek-ruzyczka@swps.edu.pl

**Keywords:** psychiatric nurses, resilience, perceived stress

## Abstract

**Background**: Resilience plays a fundamental role in the professional functioning of psychiatric nurses, supporting coping with stress, adaptation to demanding work environments, and job satisfaction. Regular assessment of resilience and perceived stress is important for timely psychological and organizational support. This study aimed to examine the relationship between resilience and perceived occupational stress among psychiatric nursing staff. **Methods**: The present study employed a cross-sectional research design. Standardized psychometric instruments were used, including the Resilience Assessment Scale (SPP-25) and the Perceived Stress Scale (PSS-10). Additionally, a self-developed questionnaire was administered to gather sociodemographic data. The study was conducted in five psychiatric hospitals located in the southern region of Poland. Data collection took place between January and June 2023 and involved a total of 555 nurses (449 women and 106 men) employed in inpatient psychiatric wards. A statistical significance level of *p* < 0.05 was adopted. Statistical analyses were performed using IBM SPSS Statistics 25. **Results**: The overall resilience level in the study group was 57.83 points (SD = 24.33). The mean level of perceived stress among psychiatric nurses was 18.43 points (SD = 10.91). Both resilience and perceived stress levels varied significantly depending on selected sociodemographic and occupational characteristics. A statistically significant negative correlation was found between perceived stress and both the overall level of resilience and each of its individual components (*p* < 0.001). **Conclusions**: Regular assessment of psychological resilience and stress levels among psychiatric nurses is an essential component of monitoring their well-being. Such evaluations provide insights into the role of individual resources in coping with occupational demands and serve as a foundation for developing targeted support programs. Strengthening resilience not only promotes the mental health of nursing staff but also contributes to higher quality of patient care and greater effectiveness in fulfilling professional responsibilities.

## 1. Introduction

Psychiatric nursing is recognized as one of the most psychologically demanding areas of healthcare. Nurses working in psychiatric wards are routinely exposed to patients exhibiting aggressive, self-harming, or suicidal behaviors, which significantly increases occupational stress and emotional burden. Research consistently demonstrates that psychiatric nurses experience higher stress levels and burnout rates compared to nurses in other medical specialties, leading to increased absenteeism, turnover intentions, and reduced quality of patient care [1,2,3,4,5,6]. Identifying factors that mitigate stress and support psychological well-being in this population is therefore essential for sustaining the mental health workforce and ensuring patient safety.

The concept of “resilience” was introduced into scientific literature in the second half of the 20th century by American psychologists Jack and Jeanne Block. Their research focused on self-control and social approval. The findings demonstrated that individuals who exhibit independence in these areas possess a strong sense of identity, adapt quickly to changing everyday conditions, and are able to cope harmoniously with encountered stressors. Moreover, their life experiences do not significantly alter their overall life orientation [7,8]. Although Jack and Jeanne Block first introduced the concept of psychological resilience into the scientific literature, subsequent researchers have broadened its meaning and applications. For instance, Emmy Werner, through her seminal longitudinal studies of children in Hawaii, demonstrated that some individuals can function adaptively despite severe adversity, emphasizing the importance of protective factors and supportive environments [9]. Similarly, Boris Cyrulnik, a French psychiatrist and psychoanalyst, extended the concept to adults, focusing on the psychological mechanisms that enable recovery and growth following trauma [10]. Together, these contributions shaped resilience into a multidimensional construct encompassing emotional, cognitive, and social processes that promote adaptation under stress.

Resilience is a factor determining an individual’s ability to adapt rapidly in the face of changing life circumstances. It enables the effective mobilization of personal competencies and environmental resources, thereby facilitating efficient functioning during exposure to both internal and external stressors. Here, “stressors” refer to environmental or situational demands that challenge the individual, whereas “perceived stress” denotes the subjective experience of strain or tension in response to these demands. Resilience and coping strategies represent the individual’s capacity to manage or mitigate the impact of stressors, promoting psychological well-being [7,11,12,13,14,15,16,17].

A high level of resilience enhances effective stress management, which in turn supports both psychological and physical well-being. Resilience influences the primary appraisal of stressful situations, making them less likely to be perceived as real threats due to the characteristic tendency toward positive emotionality. This, in turn, leads to the selection of effective coping strategies [7,8,11,15,16,17]. The positive emotionality typical of resilient individuals helps to develop psychological, physical, and social resources, thereby enabling effective coping with burdensome life events [17,18,19]. Individuals with a high level of resilience tend to adapt more easily to changes, explore various solutions, and recover more quickly to a state of psychological equilibrium [7,8,11].

Stress is a phenomenon analyzed in numerous theoretical frameworks, each offering different perspectives depending on the scientific discipline. These perspectives provide complementary insights into the causes, mechanisms, and consequences of stress, underscoring its complex and multidimensional nature.

One of the most influential psychological models of stress is the transactional model proposed by American psychologists Richard Lazarus and Susan Folkman. According to this model, stress arises when an individual perceives that environmental demands exceed their available resources, thereby threatening their well-being. The model is described as transactional to emphasize the ongoing interaction between the person and their environment—a dynamic process rather than a static event [19,20,21].

This interaction is shaped by cognitive appraisal, through which an individual evaluates whether a situation is potentially stressful (primary appraisal) and whether they possess sufficient coping resources (secondary appraisal). Because appraisal is inherently subjective, the same situation may be experienced as stressful, beneficial, or neutral depending on personal interpretation. Stress occurs when perceived demands are judged to exceed one’s coping capacity, activating physiological and psychological responses [20,21].

Another prominent framework, the Conservation of Resources (COR) theory, developed by Stevan Hobfoll, approaches stress from a resource-based perspective. Formulated in the 1980s, it integrates interdisciplinary, cultural, and evolutionary viewpoints to explain stress and coping mechanisms. Hobfoll posited that individuals strive to obtain, maintain, and protect resources they value—including material possessions, personal characteristics, conditions, and energy. Stress arises in three primary contexts: (1) actual resource loss, (2) threat of resource loss, or (3) insufficient resource gain following investment. Importantly, people differ in their ability to acquire and preserve these resources, which explains variability in stress resilience and coping effectiveness [22].

These theoretical approaches highlight the critical role of personal resources—such as resilience—in determining how individuals appraise and respond to stressful situations. This is particularly relevant in the context of psychiatric nursing, where professionals are continuously exposed to complex emotional and interpersonal demands. Nurses working in psychiatric settings frequently care for patients with suicidal tendencies, aggression, or behaviors that pose safety risks to both patients and staff [1,2,3]. Consequently, psychiatric nurses experience substantially higher levels of occupational stress and emotional burden compared with colleagues in other clinical departments [4,5,6].

Resilience plays a fundamental role in the work of nursing staff in inpatient psychiatric wards, serving as an essential factor in coping with difficult experiences, adapting to demanding conditions, and maintaining high levels of job satisfaction. Simultaneously, resilience has a significant impact on preserving nurses’ mental health and their professional efficacy [6,23,24,25].

Higher levels of resilience have been observed among psychiatric nurses [26]. This ability is associated with the need for rapid adaptation to highly demanding and stressful work environments. A high level of resilience enables psychiatric nurses to function effectively in challenging situations, maintain an empathetic attitude toward patients, and remain consistently engaged in their professional responsibilities. Resilience fosters a positive outlook, emotional regulation, and flexibility in responding to workplace challenges [6,27,28,29,30].

A review of the scientific literature on the measurement of resilience and stress intensity among psychiatric nurses in Poland reveals notable gaps in research. The few existing studies assessing resilience levels rarely focus specifically on nurses employed in psychiatric settings. Furthermore, available publications emphasize the stress-inducing characteristics of psychiatric work, while direct evaluations of stress levels are infrequent and often limited to small, non-representative samples, complicating the comparability of findings.

It remains essential to determine the level of personal resources—particularly resilience—among nurses working in psychiatric wards in Poland. This will allow for an assessment of the extent to which such resources may mitigate occupational stress. Understanding this relationship is critical for both the prevention of professional burnout and the development of effective interventions to support the mental well-being of nursing personnel. In light of increasing professional demands and staffing shortages in healthcare, identifying factors that promote psychological resilience is particularly important to ensure the continuity and quality of psychiatric care.

The present study is theoretically grounded in the transactional model of stress and the Conservation of Resources (COR) theory. According to the transactional model, stress results from a dynamic interaction between the individual and environmental demands, mediated by cognitive appraisal processes. Resilient individuals are more likely to perceive potential stressors as manageable, selecting effective coping strategies that prevent excessive emotional strain. Similarly, the COR theory emphasizes the importance of personal resources—such as resilience—in managing stress. Stress occurs when resources are threatened or lost, or when investments in resources fail to yield expected gains. By assessing resilience, the present study evaluates a personal resource that can buffer the impact of occupational stress in psychiatric nurses.

The aim of the present study was to assess the relationship between the level of resilience and the intensity of perceived stress among psychiatric nurses. Our hypothesis was that the level of resilience would be negatively associated with the perceived stress experienced by psychiatric nurses.

## 2. Materials and Methods

### 2.1. Study Design and Data Collection

The study had a cross-sectional design. It employed the estimation method and standardized scaling technique using scale sheets, as well as a diagnostic survey method through the questionnaire technique [31,32].

The selection of facilities for the study was purposive, motivated by the desire to include psychiatric wards with diverse characteristics to ensure greater representativeness and a broader research perspective. Prior to commencing research activities, written consent was obtained from the facility directors to conduct the study. Requests for consent were sent to eight psychiatric hospitals in southern Poland. Directors of three hospitals declined participation.

Participant selection was purposive. The study group was homogeneous, consisting of individuals employed as nurses in inpatient psychiatric wards who met the inclusion criteria. The inclusion criteria were: consent to participate and employment as a nurse in inpatient psychiatric wards. Exclusion criteria included employment in inpatient psychiatric wards in positions other than nursing staff, despite having nursing qualifications, and lack of consent to participate. The study encompassed several types of inpatient psychiatric wards, including, among others, general adult, geriatric, addiction, child and adolescent, forensic, and intensive care units, thereby ensuring the inclusion of nurses from diverse psychiatric care settings. No control group was identified in the study design.

The study was conducted in inpatient psychiatric wards across five hospitals located in the Lesser Poland and Silesia regions in southern Poland. Hospitals from southern Poland were selected because this region includes several large psychiatric centers with diverse institutional profiles, providing access to a varied and representative sample of psychiatric nurses. The study began in January 2023 and concluded in June 2023.

The research procedure involved a single stage. After receiving information about the purpose, scope, and nature of the project, providing informed consent, and meeting the inclusion criteria, participants completed the provided scale sheets and questionnaires independently in their workplace. Paper questionnaires were distributed to nurses employed in the participating psychiatric wards, and completed forms were placed in sealed envelopes and returned to the researchers to ensure anonymity and prevent duplicate responses. Before completing the questionnaires, participants were instructed to respond with reference to their professional experiences and feelings related to their work as psychiatric nurses, rather than their general life circumstances.

All nurses employed in the participating inpatient psychiatric wards were invited to participate in the study. The number of distributed questionnaires was proportional to the size of the nursing staff at each hospital. The overall participation rate ranged from approximately 63% to 73% across the different sites. In total, 593 nurses consented to participate; however, only 555 fully completed questionnaires were included in the final statistical analysis, as incomplete responses were excluded. Due to the anonymized nature of the data, it was not possible to compare characteristics between respondents and non-respondents. No a priori sample size calculation was performed because the study aimed to include all nurses employed in the participating psychiatric hospitals. This comprehensive inclusion approach ensured maximum coverage of the target population.

The study was conducted and reported in accordance with the Strengthening the Reporting of Observational Studies in Epidemiology (STROBE) guidelines for cross-sectional research.

### 2.2. Measurement Tools

The study utilized standardized research instruments: the Resilience Assessment Scale (SPP-25), the Perceived Stress Scale (PSS-10) and a self-designed questionnaire to collect sociodemographic data.

Resilience Assessment Scale (SPP-25), developed by Nina Ogińska-Bulik and Zygfryd Juczyński, enables the assessment of overall resilience and its five components. The questionnaire consists of 25 statements reflecting various personality traits that constitute resilience. The five components of the scale are: “Persistence and determination in action”, “Openness to new experiences and sense of humour”, “Personal competence in coping and tolerance of negative emotions”, “Tolerance of failures and treating life as a challenge”, and “Optimistic attitude toward life and the ability to mobilize in difficult situations.” Each item is rated on a 5-point Likert scale (1—strongly disagree to 5—strongly agree). Item scores are summed to yield a total score ranging from 0 to 100, with higher scores indicating greater resilience. Scores can also be calculated separately for each of the five components, each ranging from 0 to 20 points. The overall result may additionally be expressed on a standardized sten scale (1–10), where scores of 1–4 indicate low resilience, 5–6 average resilience, and 7–10 high resilience. Sten interpretations are not available for the individual components, but their scores can be used to provide a preliminary assessment of resilience in specific areas.

The internal consistency of the overall Resilience Assessment Scale, assessed using Cronbach’s alpha, was 0.89. Cronbach’s alpha coefficients for the five subscales ranged between 0.67 and 0.75. Internal stability, assessed by the test–retest method in a group of 30 individuals (both women and men) over a four-week interval, was 0.85 [33]. In the present study, internal consistency was high (Cronbach’s α = 0.98 for the total score and 0.84–0.90 for the five subscales), confirming good reliability in the examined sample.

Perceived Stress Scale (PSS-10), developed by Sheldon Cohen, Tom Kamarck, and Robin Mermelstein, was adapted into Polish by Zygfryd Juczyński and Nina Ogińska-Bulik. The scale measures perceived stress intensity and consists of ten items that assess feelings related to current problems, personal events, and coping strategies. Participants select one of five response options that best describe their feelings in the present situation (0—“never,” 1—“almost never,” 2—“sometimes,” 3—“fairly often,” 4—“very often”). Item scores are summed to yield a total score ranging from 0 to 40, with higher scores indicating greater perceived stress.

The internal consistency of the Polish adaptation was verified in a study of 120 adults, yielding a Cronbach’s alpha of 0.86. Scale reliability was further confirmed in a test–retest study of 30 students, with coefficients of 0.90 over a two-day interval and 0.72 over four weeks [34]. In the present study, internal consistency of the PSS-10 was high (Cronbach’s α = 0.95), confirming satisfactory reliability in the examined sample.

The self-designed questionnaire included questions regarding gender, age, place of residence, education level, specializations, length of nursing experience, professional experience in psychiatric wards, type of psychiatric ward currently employed in, current employment fraction, work schedule, managerial functions, marital status, and parenthood. The questions were either closed-ended or semi-open.

### 2.3. Data Analysis

Statistical analysis of the collected data was conducted using IBM SPSS Statistics 25 software.

Data related to dependent and independent variables were presented by calculating basic descriptive statistics: counts (n) and percentages (%). For quantitative variables, the mean (M), standard deviation (SD), median (Me), minimum (min) and maximum (max) values, as well as measures of distribution shape—skewness (Sk) and kurtosis (Kurt)—were calculated.

Parametric tests, including Pearson’s correlation analysis, and non-parametric tests were used to examine relationships between variables. To evaluate the normality of quantitative variables, Kolmogorov–Smirnov tests were initially performed. For variables showing statistically significant deviations, skewness and kurtosis were further assessed. Following widely accepted guidelines, values within the range of ±2 were considered acceptable for the application of parametric tests. Based on these assessments, parametric tests were applied when appropriate, while non-parametric tests were used for variables not meeting these assumptions.

A significance level of *p* < 0.05 was adopted for the study.

### 2.4. Ethical Considerations

On 12 October 2022, the research project received positive approval from the Bioethics Committee of Jagiellonian University (approval number: 1072.6120.213.2022). Prior to completing the questionnaires, all participants provided written informed consent. They received detailed information about the study objectives, the voluntary nature of participation, data confidentiality, and their right to withdraw at any time without any negative consequences. Participants did not receive any compensation for their involvement. The project and the conduct of the study adhered to the principles of Good Scientific Practice [35] and the Declaration of Helsinki [36].

## 3. Results

### 3.1. Sociodemographic Characteristics of Respondents

The responses of 555 participants were analyzed, including 449 women (80.9%) and 106 men (19.1%). The age of the respondents ranged from 22 to 62 years (M = 43.07; SD =11.65). Their work experience in psychiatric wards ranged from six months to 43 years (M = 17.14; SD = 11.76), while total professional experience in nursing ranged from 1 to 43 years (M = 20.23; SD = 12.39). The remaining sociodemographic data of the respondents are presented in Table 1.

### 3.2. Resilience Level of Psychiatric Nurses

The overall level of resilience among psychiatric nurses ranged from 14 to 88 points. The mean general resilience score in the study group was 57.83 points (SD = 24.33), with a median of 61 points. The obtained total score corresponds to sten 3, indicating a low level of psychological resilience. Although sten scores are not available for the individual subscales of the Resilience Assessment Scale, the results allow for a preliminary interpretation of resilience as ranging from low to moderate. Among the subscales, the highest mean values were observed for Openness to new experiences and sense of humour (M = 12.22), Tolerance of failures and treating life as a challenge (M = 11.85), and Persistence and determination in action (M = 11.76), suggesting that these aspects of resilience are relatively more developed in the studied group. Detailed results for all subscales are presented in Table 2.

The analysis revealed significant relationships between the overall level of resilience and several sociodemographic and occupational variables. Resilience was significantly higher among men compared to women (U = 17,250.0, Z = −3.86, *p* < 0.001) and among nurses with higher education or completed specializations (H = 168.40, *p* < 0.001; H = 23.14, *p* < 0.001). In contrast, resilience decreased with increasing age (r = −0.57, *p* < 0.001) and longer professional experience both in psychiatric wards (r = −0.51, *p* < 0.001) and in nursing overall (r = −0.56, *p* < 0.001). Nurses living in larger cities exhibited higher resilience than those residing in smaller towns or rural areas (H = 78.14, *p* < 0.001). Differences were also observed according to the type of psychiatric ward (H = 24.13, *p* = 0.012), with the highest resilience reported by nurses working in forensic and child/adolescent units. Regarding personal factors, married nurses and those with children showed lower resilience compared to unmarried or childless respondents (*p* < 0.001). A weak negative correlation was also noted between resilience and the number of children (r = −0.14, *p* = 0.006). No statistically significant relationships were found between resilience level and work–time ratio, work arrangement, or managerial position. The remaining detailed results for selected analyzed sociodemographic and occupational variables are presented in Table 3.

### 3.3. Level of Perceived Stress Among Psychiatric Nurses

The perceived stress level of nurses employed in inpatient psychiatric wards ranged from 1 to 40 points, with the most frequent scores falling between 4 and 9 points. In the study group, the median score was 18 points. The mean score was 18.43 points (SD = 10.91) (Table 4), corresponding to sten 6, which indicates a moderate level of perceived stress.

The analysis revealed significant relationships between perceived stress levels and several sociodemographic and occupational variables. Perceived stress was significantly higher among women compared to men (U = 17,354.5, Z = −3.79, *p* < 0.001) and among nurses with secondary education compared to those with higher degrees (H = 199.74, *p* < 0.001). Stress levels were also higher among nurses without a nursing specialization or still in training compared to those who had completed one (H = 19.37, *p* < 0.001). In contrast, perceived stress increased with age (r = 0.66, *p* < 0.001), length of service in psychiatric wards (r = 0.58, *p* < 0.001), and overall work experience (r = 0.64, *p* < 0.001). Nurses living in rural areas reported the highest stress levels, whereas those residing in large cities (>250,000 inhabitants) showed the lowest stress (H = 60.95, *p* < 0.001). Significant differences were also observed depending on the type of psychiatric ward (H = 24.64, *p* = 0.020), with stress levels being highest in general and chronic psychiatric wards and lowest in rehabilitation, forensic, and child/adolescent units. Regarding personal characteristics, married nurses (H = 65.23, *p* < 0.001) and those with children (U = 24,908.0, Z = −3.99, *p* < 0.001) reported higher perceived stress. A weak positive correlation was also found between the number of children and stress level (r = 0.12, *p* = 0.018). No statistically significant relationships were found between perceived stress level and work-time ratio, work arrangement, or managerial position. The remaining detailed results for selected analyzed sociodemographic and occupational variables are presented in Table 5.

### 3.4. The Relationship Between the Level of Resilience and the Intensity of Stress Experienced by Psychiatric Nurses

The obtained results allowed for the identification of a relationship between the overall level of resilience and its individual components, and the intensity of perceived stress among nurses employed in psychiatric wards. The level of perceived stress was negatively correlated with overall resilience as well as with all of its components (*p* < 0.001) (Table 6).

## 4. Discussion

This study focused on psychiatric nurses working in inpatient mental health wards, a professional group exposed to unique and intense occupational stressors. A review of the existing scientific literature indicates that the issue of psychological resilience among psychiatric nurses has been largely overlooked in Polish research, despite being addressed more consistently in international studies. Many publications focus on identifying factors that influence resilience and on strategies for enhancing it. It is important to note that comparing research findings related to resilience measurement is challenging due to the variety of assessment tools employed. This diversity may result in inconsistencies in data interpretation and hinder the formulation of clear and generalizable conclusions [24,37].

In the present study, the overall level of resilience among psychiatric inpatient nursing staff ranged from low to moderate. One of the most commonly used instruments for measuring resilience is the Connor–Davidson Resilience Scale (CD-RISC), developed by Kathryn Connor & Jonathan Davidson. Its structure is comparable to the Resilience Measurement Scale by Ogińska-Bulik, used in this study. Both tools consist of 25 items rated on a 4-point scale, which indicates a similar construction and methodology for assessing psychological resilience. Furthermore, the scoring range for the overall resilience level is identical, allowing for partial comparison of results [32,38]. This tool has been employed in studies assessing resilience levels in psychiatric nurses, as referenced below [39,40,41,42].

Few researchers have undertaken an analysis of resilience in the context of the work environment. In the study by Foster et al., which included 498 Australian psychiatric nurses, the impact of occupational stressors on mental health was investigated. The results indicated that staff resilience, measured using the 23-item Workplace Resilience Inventory, was at a moderate level. The resilience of psychiatric nurses was assessed across three dimensions of this scale: emotional self-regulation processes (M = 3.3, SD = 0.6), behavioral self-regulation processes (M = 3.2, SD = 0.5), and cognitive self-regulation processes (M = 3.1, SD = 0.8), each reflecting a moderate level of resilience [6]. In turn, the study by Delgado et al., which examined psychological well-being, stress levels, and resilience among Australian psychiatric nurses, utilized the 25-item Resilience at Work questionnaire. Among the 482 study participants, more than half demonstrated a high level of resilience (M = 70.27) [42]. Despite the challenges in comparing results due to the use of different research instruments across the aforementioned studies, it can be observed that the overall level of psychological resilience among psychiatric nurses—regardless of country of origin—remains at a varied level [38,39,40,41,42].

The results of the present study showed that the overall level of psychological resilience among nurses working in psychiatric wards was significantly associated with selected sociodemographic and occupational variables. A higher level of resilience was observed among men, which may indicate greater ability to cope with emotional burdens or different patterns of stress response among men working in nursing. Previous studies have not provided consistent findings in this regard—Sukut et al. [40] and Dogan and Boyacioglu [43] did not confirm gender differences in resilience, which may be due to cultural factors, sample size, or specific work environments. The present study also found that increasing age and longer professional experience, both overall and in psychiatric wards, were associated with lower resilience. This may reflect the gradual depletion of personal resources as a result of prolonged exposure to occupational stress and emotional strain, as also noted by Delgado et al. [42]. Conversely, Foster et al. [6] reported that younger nurses had lower resilience levels, possibly due to limited experience and less developed coping strategies. In the present study, nurses living in larger cities demonstrated higher resilience, which may result from better access to social support, professional training, and resources that promote competence development. In contrast, lower resilience among nurses living in rural areas may be linked to greater social isolation and fewer opportunities for professional advancement. Education level and specialization in nursing also emerged as important factors differentiating resilience. Nurses with higher education and completed specialization exhibited greater resilience, highlighting the role of education as a protective factor that fosters adaptive skills and psychological strength [44]. These results partly differ from those of Sukut et al. [40], who found higher resilience among nurses with secondary education, but they align with the general view that education supports the development of effective stress-coping strategies. Differences were also observed depending on the type of psychiatric ward. The highest levels of resilience were found among nurses working in forensic and child/adolescent psychiatric units, which may be related to the need for frequent decision making in crisis situations and the emotionally demanding nature of the work, requiring greater psychological flexibility. Furthermore, the analysis showed that nurses who were married exhibited lower resilience compared with unmarried individuals. This may stem from additional family responsibilities and difficulties in maintaining work–life balance, as confirmed by previous studies suggesting that domestic stress can negatively affect resilience [39]. Similarly, nurses with children demonstrated slightly lower resilience than childless participants, and resilience decreased further with the increasing number of children. This may be explained by reduced time for recovery and limited access to resources supporting psychological well-being.

Scientific literature has documented the effectiveness of interventions aimed at enhancing and developing resilience among psychiatric nurses. Henshall, Davey & Jackson conducted an intervention program for British psychiatric nurses that focused on strengthening skills in positive and critical thinking, achieving work–life balance, and fostering inner spiritual development. The primary aim of the intervention was to enhance and intensify adaptive capacities in the area of psychological resilience. The program was based on a mentoring relationship model. A total of 29 psychiatric nurses and 22 mentors participated in the study. The results indicated that, following the intervention, participants reported an increase in psychological resilience and a greater awareness of its importance. Additionally, the nurses demonstrated increased self-reflection regarding their personal resilience development process [27].

Implementing resilience-building programs may contribute to improved self-confidence, increased job satisfaction, and stronger interpersonal relationships among staff, while also enhancing the quality of patient care. The development and implementation of such programs and training initiatives aimed at strengthening resilience in nursing personnel should be the subject of further research.

A review of the available literature indicates that the assessment of perceived stress intensity has been relatively well explored in scientific research. Numerous publications focus on identifying the factors influencing stress experienced by psychiatric nurses and on seeking effective strategies to alleviate it [45].

In the present study, the mean perceived stress level was 18.43 points, which, according to the theoretical assumptions of the measurement tool, can be interpreted as a moderate level of stress intensity. In a study conducted by Masa’Deh, Jarrah, and AbuRuz among 163 Jordanian psychiatric nurses, the mean score of perceived stress was 27.57 points, indicating a high level of stress [46]. In the study by Shahrour et al., which aimed to determine whether social support moderates the relationship between stress and workplace violence among 195 psychiatric nurses, the average perceived stress level measured by the PSS-10 scale was 21.03 points [47]. The study conducted by Zarvijani, Moghaddam & Parchebafieh aimed to evaluate the impact of acceptance and commitment therapy on the level of stress perceived by psychiatric nurses. The sample included 70 participants—35 nurses were assigned to the experimental group and 35 to the control group. Before the intervention, the average stress level was 23.42 points (SD = 6.02) in the experimental group and 23.36 points (SD = 6.38) in the control group, indicating a moderate level of stress in both groups [48]. The reported findings demonstrate relative consistency, allowing for the conclusion that psychiatric nurses tend to experience moderate to high levels of perceived stress.

The results of this study demonstrated that the level of perceived stress among psychiatric nurses varied according to selected sociodemographic and occupational characteristics. Women reported higher stress levels than men, consistent with the findings of Yada et al. [49] and Agyemang et al. [50]. This may be related to women’s greater exposure to aggressive or violent behaviors from patients in psychiatric settings, as well as to gender differences in emotional responses and coping strategies. Stress levels increased with age and with longer professional experience, both overall and in psychiatric wards. This trend may reflect the cumulative impact of prolonged exposure to emotional strain, gradual depletion of psychological resources, and limited opportunities for recovery. These results align with the findings of Yao et al. [51] and Eita and Alhalawany [52], who also observed a positive relationship between years of work experience and perceived stress among psychiatric nurses. Nurses residing in rural areas reported higher stress levels than those living in urban settings. This may be attributed to limited access to professional psychological support, fewer opportunities for continuing education and peer collaboration, and a greater sense of social isolation. Education emerged as an important protective factor. Participants with higher education and completed nursing specializations demonstrated lower levels of perceived stress, likely due to enhanced cognitive and interpersonal competencies that facilitate more effective coping with occupational demands. These findings are consistent with those of Agyemang et al. [50]. Differences in perceived stress were also observed across psychiatric ward types. The highest stress levels were reported among nurses working in general and chronic psychiatric wards, which may be related to prolonged exposure to patient aggression and limited possibilities for therapeutic intervention. Marital status and parenthood were also associated with stress intensity. Nurses who were married reported higher stress levels, possibly due to the combined burden of professional and family responsibilities and difficulties maintaining work–life balance. Conversely, having children was associated with lower stress levels, potentially reflecting the emotional support and sense of purpose derived from parenthood; however, stress increased again with a greater number of children, likely due to reduced time for rest and recovery.

Overall, these findings confirm that occupational stress among psychiatric nurses is a multidimensional phenomenon shaped by both individual and contextual factors. Understanding these relationships is essential for designing targeted interventions aimed at reducing stress and strengthening psychological resources in this professional group.

The available literature confirms the effectiveness of interventions aimed at reducing stress among psychiatric nurses [53]. An experimental study by Mao et al., conducted on a group of 150 psychiatric nurses (75 in the experimental group and 75 in the control group), demonstrated that Balint group sessions can effectively reduce levels of stress, anxiety, and depressive symptoms while also enhancing empathy among nursing staff working in psychiatric wards [54].

Mindfulness-Based Stress Reduction (MBSR) practices have also proven effective in alleviating stress experienced by psychiatric nurses. In an experimental study by Yang, involving 100 psychiatric nurses (50 in the experimental group and 50 in the control group), MBSR therapy significantly contributed to reducing stress levels [55]. Similarly, a study by Zaki & Barakat, conducted with 40 psychiatric nurses, showed that stress reduction training—including regular sessions aimed at developing skills such as team communication, problem solving, and time management—effectively lowered stress levels [56]. These results support the implementation of interventions targeting stress reduction among psychiatric nurses.

The present study demonstrated a relationship between the overall level of resilience—as well as its five components—and the intensity of perceived stress among nurses working in inpatient psychiatric wards. Lower levels of resilience among respondents were associated with higher levels of perceived stress. This inverse relationship is consistent with the Transactional Model of Stress by Lazarus and Folkman, as well as the Conservation of Resources theory proposed by Hobfoll, which emphasizes resilience as a crucial protective factor mitigating the impact of stressors.

A study conducted by Chen et al., involving 413 psychiatric nurses, aimed to assess the mediating role of resilience in relation to perceived stress levels and mental health. The results indicated that higher resilience was associated with lower perceived stress among participants [57]. Similar findings were reported by Cabrera-Aguilar et al., who in a study of 459 nurses found that higher resilience correlated with lower stress levels [58]. These observations were further supported by research conducted by Khanmohammadi et al., involving 150 Iranian nurses, which demonstrated that higher resilience levels resulted in lower perceived stress [59].

This study should be considered in terms of its strengths and limitations. A major strength lies in its in-depth exploration of psychological resilience and its relationship with stress levels among psychiatric nurses, a topic rarely addressed in Polish scientific literature. The theoretical foundations of the study ensure consistency with existing research, enhancing the reliability and validity of the results. The use of standardized research instruments allows for replication in multicenter settings and, with randomized sampling, enables broader comparisons within the population of psychiatric nurses.

Among the study’s limitations are its cross-sectional design, which precludes causal inferences, and the use of purposive rather than random sampling, involving only five hospitals in southern Poland. The relatively small sample size and geographic restriction may limit the representativeness of the findings; therefore, caution is warranted when generalizing the results to the wider population of psychiatric nurses. An a priori sample size calculation was not conducted, which should be considered an additional methodological limitation. Compared to international studies, the present findings provide region-specific evidence, which can be valuable for future cross-cultural comparisons. Moreover, employing random sampling or stratification in future studies could improve external validity, while including nurses from other hospital wards or non-psychiatric departments might yield valuable comparative data. The study did not include potential mediating mechanisms (e.g., resilience as a mediator between stressors and coping outcomes), which have been highlighted in previous literature, thus limiting the analytic depth of the findings. Another limitation concerns the psychometric tools. Although both the SPP-25 and PSS-10 scales demonstrate good reliability, they remain self-report measures and may be subject to response biases. Their validation was conducted on specific populations, which may limit generalizability to the current sample. It is also worth acknowledging cultural and systemic factors that might explain why Polish psychiatric nurses, in contrast to their counterparts in some international studies, tend to report low resilience alongside moderate levels of perceived stress. Finally, although the study addressed sensitive topics related to occupational stress, the use of an anonymous and voluntary questionnaire format minimized potential emotional risks for participants, and no adverse effects were anticipated. Nevertheless, given the sensitive nature of the subject matter, future research should consider incorporating optional support mechanisms for participants to ensure their well-being while reflecting on occupational stress experiences.

### Implication for Practice

Resilience may serve as a protective buffer in work environments characterized by high emotional demands, contributing to the maintenance of mental well-being. Regular monitoring of resilience levels and occupational stress intensity among psychiatric nurses can enable early identification of at-risk groups and precise tailoring of supportive interventions. Assessing these variables can provide essential data for planning preventive measures and development programs that could be implemented both at individual and institutional levels.

The findings of the present study hold significant practical implications. Preventing chronic stress among nursing staff employed in inpatient psychiatric wards requires conscious strengthening of their personal resources, including psychological resilience. These personal resources may function as moderating factors in coping with stress. As integral components of professional competencies, they support effective functioning in challenging situations, provide protection in demanding, high-stress work environments, and contribute to ensuring safe and professional patient care.

Therefore, it is essential to intensify the development and implementation of training programs and workshops aimed at enhancing psychological resilience among nurses working in psychiatric inpatient units. Such initiatives should be conducted both as part of postgraduate education and in-house training, led by experienced supervisors. These programs could include components such as stress management techniques, mindfulness and relaxation practices, development of coping strategies, emotional self-regulation exercises, and peer-support or mentoring systems. In addition, practical guidance on integrating resilience skills into daily nursing practice—tailored to the specific organizational and cultural context of Polish psychiatric care—should be provided. While similar resilience-building interventions have been evaluated internationally, future research should explore their adaptation and effectiveness in local settings to ensure relevance, feasibility, and measurable impact on both staff well-being and quality of patient care.

## 5. Conclusions

The analysis of the findings allows several conclusions to be drawn. Nurses employed in inpatient psychiatric wards exhibited resilience levels ranging from low to moderate, accompanied by a moderate intensity of perceived stress. Moreover, higher resilience was associated with lower perceived stress. Individual and occupational characteristics were also found to significantly influence both variables. Lower resilience and higher stress were observed among women, older nurses, and those with longer professional experience. In contrast, higher education, completed nursing specialization, and employment in forensic or child and adolescent wards were associated with greater resilience and lower stress levels.

Regular monitoring of resilience levels and occupational stress among psychiatric nurses may enable early identification of at-risk groups and precise tailoring of supportive interventions. Assessing these variables can provide essential data for planning preventive measures and development programs that could be implemented both at individual and institutional levels.

## Figures and Tables

**Table 1:** Sociodemographic characteristics of respondents.

		n	%
Place of residence	Village	210	37.8
	City < 250 thousand inhabitants	172	31.0
	City > 250 thousand inhabitants	173	31.1
Level of education	Secondary vocational education	163	29.4
	Bachelor’s degree	147	26.5
	Master’s degree	236	42.5
	Doctoral degree/academic title	9	1.6
Specialization in the field of nursing	No specialization	202	36.4
	In progress	99	17.8
	Completed (including seven individuals currently pursuing a second specialization)	254	45.8
Work–time ratio in psychiatric wards	0.25	4	0.7
	0.5	41	7.5
	0.75	5	0.9
	1	489	88.0
	1.25	1	0.2
	1.5	13	2.3
	2	2	0.4
Work–time ratio in other wards	0	380	68.5
	0.25	15	2.7
	0.33	4	0.7
	0.5	101	18.2
	0.75	8	1.4
	1	42	7.6
	1.5	5	0.9
Type of psychiatric ward	General psychiatric ward	130	23.4
	Child and adolescent psychiatry ward	29	5.2
	Basic security forensic psychiatry ward	61	11.0
	Enhanced security forensic psychiatry ward	64	11.5
	Enhanced security forensic psychiatry ward for juveniles	12	2.2
	Psychiatric rehabilitation ward	40	7.2
	Psychogeriatric ward	46	8.3
	Alcohol detoxification ward	40	7.2
	Alcohol addiction treatment ward	58	10.5
	Psychoactive substance addiction treatment ward	18	3.2
	Personality and neurotic disorders treatment ward	13	2.3
	General psychiatry ward with basic security forensic psychiatry subunit	18	3.2
	Long-term psychiatric care ward	19	3.4
	More than one ward	7	1.3
Work arrangement	Day shift	81	14.6
	Rotating shifts	474	85.4
Management position	Yes	58	10.5
	No	497	89.5
Marital status	Single	114	20.6
	Informal relationship	75	13.6
	Married	278	50
	Divorced	60	10.8
	Widowed	28	5.0
Parental status	Yes	386	69.6
	No	169	30.4
Number of children	1	141	25.4
	2	154	27.7
	3	57	10.3
	4	21	3.8
	5	6	1.1
	6	3	0.5

n—number of individuals; %—percentage.

**Table 2:** Level of resilience of psychiatric nurses.

Resilience—Resilience Assessment Scale (SPP-25)	M	Me	SD	Sk.	Kurt.	Min	Max	D	*p*
Overall level of resilience	57.83	61	24.33	−0.46	−1.24	14	88	0.16	<0.001
Persistence and determination in action	11.76	13	5.17	−0.54	−0.87	0	19	0.13	<0.001
Openness to new experiences and sense of humour	12.22	13	4.68	−0.30	−1.26	2	20	0.15	<0.001
Personal competence in coping and tolerance of negative emotions	11.13	12	5.01	−0.40	−1.23	2	18	0.16	<0.001
Tolerance of failures and treating life as a challenge	11.85	14	5.36	−0.47	−1.06	1	20	0.16	<0.001
Optimistic attitude toward life and the ability to mobilize in difficult situations	10.85	12	5.03	−0.48	−1.14	1	18	0.16	<0.001

M—mean; Me—median; SD—standard deviation; Sk.—skewness; Kurt.—kurtosis; Min—minimum value; Max—maximum value; D—Kolmogorov–Smirnov test statistic; *p*—significance level.

**Table 3:** Relationships between resilience and selected sociodemographic and occupational characteristics of psychiatric nurses.

Sociodemographic and Occupational Variable Resilience Assessment Scale (SPP-25)	Categories/Type	Statistical Test	*p*	Interpretation
Gender	Female/Male	U = 17,250.0, Z = −3.86	<0.001	Men reported higher resilience than women.
Age	Continuous (r)	r = −0.57	<0.001	Resilience decreased with increasing age.
Place of residence	Village/<250 k/>250 k	H = 78.14	<0.001	Nurses living in larger cities showed higher resilience.
Work experience in psychiatric wards	Continuous (r)	r = −0.51	<0.001	Longer experience in psychiatry was associated with lower resilience.
Work experience in nursing	Continuous (r)	r = −0.56	<0.001	Greater overall work experience corresponded with lower resilience
Level of education	Secondary/BA/MA/Academic title	H = 168.40	<0.001	Higher education was linked to higher resilience.
Specialization in the field of nursing	None/In progress/Completed	H = 23.14	<0.001	Nurses with completed specialization reported the highest resilience.
Type of psychiatric ward	12 categories	H = 24.13	0.012	Resilience was lowest in general wards and highest in forensic and child/adolescent wards.
Marital status	Single/Informal/Married/Divorced/Widow(er)	H = 50.96	<0.001	Married nurses demonstrated lower resilience compared to others.
Parental status	Yes/No	U = 25,031.0, Z = −3.92	<0.001	Nurses without children showed higher resilience.
Number of children	Continuous (r)	r = −0.14	0.006	Resilience decreased slightly as the number of children increased.

U—Mann–Whitney U test; H—Kruskal–Wallis test; r—Pearson’s correlation coefficient; *p*—significance level.

**Table 4:** Level of perceived stress among psychiatric nurses.

Perceived Stress—Perceived Stress Scale (PSS-10)	M	Me	SD	Sk.	Kurt.	Min	Max	D	*p*
Level of perceived stress	18.43	18	10.91	0.22	−0.93	1	40	0.07	<0.001

M—mean; Me—median; SD—standard deviation; Sk.—skewness; Kurt.—kurtosis; Min—minimum value; Max—maximum value; D—Kolmogorov–Smirnov test statistic; *p*—significance level.

**Table 5:** Relationships between perceived stress level and selected sociodemographic and occupational characteristics of psychiatric nurses.

Sociodemographic and Occupational Variable Perceived Stress Scale (PSS-10)	Categories/Type	Statistical Test	*p*	Interpretation
Gender	Female/Male	U = 17,354.5, Z = −3.79	<0.001	Women reported higher perceived stress than men.
Age	Continuous (r)	r = 0.66	<0.001	Perceived stress increased with age.
Place of residence	Village/<250 k/>250 k	H = 60.95	<0.001	Nurses living in rural areas had the highest stress levels.
Work experience in psychiatric wards	Continuous (r)	r = 0.58	<0.001	Longer psychiatric experience was associated with higher stress.
Work experience in nursing	Continuous (r)	r = 0.64	<0.001	Longer professional experience corresponded with higher stress.
Level of education	Secondary/BA/MA/Academic title	H = 199.74	<0.001	Lower education was linked to higher stress.
Specialization in the field of nursing	None/In progress/Completed	H = 19.37	<0.001	Nurses with completed specialization showed lower stress.
Type of psychiatric ward	12 categories	H = 24.64	0.020	Stress was highest in general/chronic wards and lowest in rehabilitation, forensic, and child/adolescent units.
Marital status	Single/Informal/Married/Divorced/Widow(er)	H = 65.23	<0.001	Married nurses demonstrated higher stress level.
Parental status	Yes/No	U = 24,908.0, Z = −3.99	<0.001	Nurses with children reported higher perceived stress.
Number of children	Continuous (r)	r = 0.12	0.018	Stress slightly increased with the number of children.

U—Mann–Whitney U test; H—Kruskal–Wallis test; r—Pearson’s correlation coefficient; *p*—significance level.

**Table 6:** The relationship between the level of resilience and the intensity of perceived stress among the respondents.

Resilience—Resilience Assessment Scale (SPP-25)	Perceived Stress—Perceived Stress Scale (PSS-10)	
	r	*p*
Overall level of resilience	−0.81	<0.001
Persistence and determination in action	−0.76	<0.001
Openness to new experiences and sense of humour	−0.75	<0.001
Personal competence in coping and tolerance of negative emotions	−0.81	<0.001
Tolerance of failures and treating life as a challenge	−0.78	<0.001
Optimistic attitude toward life and the ability to mobilize in difficult situations	−0.81	<0.001

r—Pearson’s correlation coefficient; *p*—significance level.

## Data Availability

The original contributions presented in this study are included in the article. Further inquiries can be directed to the corresponding author.

## Abbreviations

The following abbreviations are used in this manuscript:

CD-RISC	Connor–Davidson Resilience Scale
COR	Conservation of Resources Theory
MBSR	Mindfulness-Based Stress Reduction
PSS-10	Perceived Stress Scale
SPP-25	Resilience Assessment Scale (SPP-25)

## References

[1] Bekelepi N., Martin P. (2023). Self-reported incidents of violence towards nurses working in acute psychiatric units. Curationis.

[2] Garcia-Izquierdo M., Meseguer de Pedro M., Rios-Risquez M.I., Sanchez M.I.S. (2018). Resilience as a Moderator of Psychological Health in Situations of Chronic Stress (Burnout) in a Sample of Hospital Nurses. J. Nurs. Scholarsh..

[3] Qi Y.K., Xiang Y.T., An F.R., Wang J., Zeng J.Y., Ungvari G.S., Newhouse R., Yu D.S., Lai K.Y., Ding Y.M. (2014). Nurses’ work-related stress in China: A comparison between psychiatric and general hospitals. Perspect. Psychiatr. Care.

[4] Tummers G.E., Janssen P.P., Landeweerd A., Houkes I. (2001). A comparative study of work characteristics and reactions between general and mental health nurses: A multi-sample analysis. J. Adv. Nurs..

[5] Alenezi A., McAndrew S., Fallon P. (2019). Burning out physical and emotional fatigue: Evaluating the effects of a programme aimed at reducing burnout among mental health nurses. Int. J. Ment. Health Nurs..

[6] Foster K., Roche M., Delgado C., Cuzzillo C., Giandinoto J.-A., Furness T. (2019). Resilience and mental health nursing: An integrative review of international literature. Int. J. Ment. Health Nurs..

[7] Block J.H., Block J., Collins W.A. (1980). The role of ego-control and ego-resiliency in the organization of behavior. Development of Cognition, Affect, and Social Relations.

[8] Block J., Kremen A.M. (1996). IQ and ego-resiliency: Conceptual and empirical connections and separateness. J. Personal. Soc. Psychol..

[9] Werner E.E. (1995). Resilience in Development. Curr. Dir. Psychol. Sci..

[10] Cyrulnik B. (2015). Opiekunowie i procesy rezyliencji. Stud. Porad./J. Cousellogy.

[11] Ogińska-Bulik N., Juczyński Z. (2010). Osobowość: Stres a Zdrowie.

[12] Uchnast Z. (1998). Prężność osobowa a egzystencjalne wymiary wartościowania. Rocz. Psychol..

[13] Fredrickson B.L. (2001). The role of positive emotions in positive psychology: The broaden-and-build theory of positive emotions. Am. Psychol..

[14] Letzring T.D., Block J., Funder D.C. (2005). Ego-control and ego-resiliency: Generalization of self-report scales based on personality descriptions from acquaintances clinicians, and the self. J. Reseach Personal..

[15] Rao G.P., Koneru A., Nebhineni N., Mishra K.K. (2024). Developing resilience and harnessing emotional intelligence. Indian J. Psychiatry.

[16] Schneider T.R., Lyons J.B., Khazon S. (2013). Emotional intelligence and resilience. Personal. Individ. Differ..

[17] Falewicz A. (2016). Prężność osobowości i jej rola w procesach radzenia sobie ze stresem. Stud. Koszalińsko-Kołobrzeskie.

[18] Fteiha M., Awwad N. (2020). Emotional intelligence and its relationship with stress coping style. Health Psychol. Open.

[19] Lazarus R., Folkman S. (1984). Stress, Appraisal, and Coping.

[20] Lazarus R. (1993). Coping theory and research: Past, present and future. Psychosom. Med..

[21] Lazarus R. (1986). Paradygmat stresu i radzenia sobie. Now. Psychol..

[22] Hobfoll S.E. (1989). Conservation of resources: A new attempt at conceptualizing stress. Am. Psychol. J..

[23] Bui M.V., EcInnes E., Ennis G., Foster K. (2023). Resilience and mental health nursing: An integrative review of updated evidence. Int. J. Ment. Health Nurs..

[24] Kim E.Y., Chang S.O. (2022). Exploring nurse perceptions and experiences of resilience: A meta-synthesis study. BMC Nurs..

[25] Itzhaki M., Peles-Bortz A., Kostistky H., Barnoy D., Filshtinsky V., Bluvstein I. (2015). Exposure of mental health nurses to violence associated with job stress, life satisfaction, staff resilience, and post-traumatic growth. Int. J. Ment. Health Nurs..

[26] Abram M.D., Jacobowitz W. (2021). Resilience and burnout in healthcare students and inpatient psychiatric nurses: A between-groups study of two populations. Arch. Psychiatr. Nurs..

[27] Henshall C., Davey Z., Jackson D. (2020). The implementation and evaluation of a resilience enhancement programme for nurses working in the forensic setting. Int. J. Ment. Health Nurs..

[28] Delgado C., Roche M., Fethney J., Foster K. (2021). Mental health nurses’ psychological well-being, mental distress, and workplace resilience: A cross-sectional survey. Int. J. Ment. Health Nurs..

[29] Matos P.S., Neushotz L.A., Griffin M.T., Fitzpatrick J.J. (2010). An exploratory study of resilience and job satisfaction among psychiatric nurses working in inpatient units. Int. J. Ment. Health Nurs..

[30] Prosser S.J., Metzger M., Gulbransen K. (2017). Don’t Just Survive, Thrive: Understanding How Acute Psychiatric Nurses Develop Resilience. Arch. Psychiatr. Nurs..

[31] Lenartowicz H., Kózka M. (2010). Metodologia Badań w Pielęgniarstwie.

[32] Serafin L. (2022). Badania Naukowe w Pielęgniarstwie. Ocena, Synteza i Tworzenie Dowodów Naukowych w Praktyce Pielęgniarskiej.

[33] Ogińska-Bulik N., Juczyński Z. (2008). Skala Pomiaru Prężności (SPP-25). Now. Psychol..

[34] Juczyński Z., Ogińska-Bulik N. (2009). Narzędzia Pomiaru Stresu i Radzenia Sobie ze Stresem.

[35] European Medicines Agency (2018). Guideline for Good Clinical Practice E6 (R2). https://www.ema.europa.eu/en/documents/scientific-guideline/ich-e-6-r2-guideline-good-clinical-practice-step-5_en.pdf.

[36] World Medical Association (2023). WMA Declaration of Helsinki—Ethical Principles for Medical Research Involving Human Subjects. https://www.wma.net/policies-post/wma-declaration-of-helsinki/.

[37] Hollywood L., Phillips K.E. (2020). Nurses’ resilience levels and the effects of workplace violence on patient care. Appl. Nurs. Res..

[38] Connor K.M., Davidson J.R. (2003). Development of a new resilience scale: The Connor-Davidson Resilience Scale (CD-RISC). Depress. Anxiety.

[39] Dehvan F., Kamangar P., Baiezeedy S., Roshani D., Ghanei-Gheshlagh R. (2018). The relationship of mental helth with resilience among psychiatric nurses. Nurs. Pract. Today.

[40] Sukut O., Sahin-Bayindir G., Ayhan-Balik C.H., Albal E. (2022). Professional quality of life and psychological resilience among psychiatric nurses. Perspect. Psychiatr. Care.

[41] Majrabi M.A., Hasan A.A., Alasmee N. (2021). Nurses burnout, resilience and its association with safety culture: A cross sectional study. Ment. Health Soc. Incl..

[42] Delgado C., Roche M., Fethney J., Foster K. (2020). Workplace resilience and emotional labour of Australian mental health nurses: Results of national survey. Int. J. Ment. Health Nurs..

[43] Dogan N., Boyacioglu N.E. (2021). Relationship between psychiatric nurses resilience and empathic tendencies. Clin. Exp. Health Sci..

[44] Guo Y.F., Cross W., Plummer V., Lam L., Luo Y.H., Zhang J.P. (2017). Exploring resilience in Chinese nurses: A cross-sectional study. J. Nurs. Manag..

[45] Alaween S., Alzayyay A., Odah M. (2024). Occupational Stress among Psychiatric Nurses: A Literature Review. Jordan J. Nurs. Res..

[46] Masa’Deh R., Jarrah S., AbuRuz M.E. (2018). Occupational stress in psychiatric nursing. Int. J. Afr. Nurs. Sci..

[47] Shahrour G., Taha I., Ali A.M., Alibrahim M. (2022). The moderating role of social support on workplace violence and stress among psychiatric nurses. Nurs. Forum.

[48] Zarvijani S.A.H., Moghaddam L.F., Parchebafieh S. (2021). Acceptance and commitment therapy on perceived stress and psychological flexibility of psychiatric nurses: A randomized control trial. BMC Nurs..

[49] Yada H., Abe H., Omori H., Matsuo H., Masaki O., Ishida Y., Katoh T. (2014). Differences in job stress experienced by female and male Japanese psychiatric nurses. Int. J. Ment. Health Nurs..

[50] Agyemang S., Ninnoni J.P., Enyan N.I.E. (2022). Prevelence and determinants of depression, anxiety and stress among psychiatric nurses in Ghana: A cross-sectional study. BMC Nurs..

[51] Yao X., Shao J., Wang L., Zhang J., Zhang C., Lin Y. (2021). Does workplace violence, empathy, and communication influence occupational stress among mental health nurses?. Int. J. Ment. Health Nurs..

[52] Eita L.H., Alhalawany R.M. (2021). The Relation between Clinical Competency and Perceived Psychiatric Nurses’ Job Stress. Tanta Sci. Nurs. J..

[53] Jappinen K., Ross M., Slater P., Suominen T. (2021). Connection between nurse managers’ stress form workload and overall job stress, job satisfaction and practice environment in central hospitals: A cross-sectional study. Nord. J. Nurs. Res..

[54] Mao Y., Zhang F., Wang Y., Hu Q., Lingyung F. (2024). The effect of balint practice on reducing stress, anxiety and depression levels of psychiatric nurses and improving empathy level. BMC Nurs..

[55] Yang J., Tang S., Zhou W. (2018). Effect of Mindfulness-Based Stress Reduction Therapy on Work Stress and Mental Health of Psychiatric Nurses. Psychiatr. Danub..

[56] Zaki M.M., Barakat M.M. (2018). Effect of Stress Management on Job Related Stress among Nurses Working with Psychiatric Patients. J. Nurs. Health Sci..

[57] Chen S.Y., Yan S.R., Zhao W.W., Gao Y., Zong W., Bian C., Cheng Y., Zhang Y.H. (2022). The mediating and moderating role of psychological resilience between occupational stress and mental health of psychiatric nurses: A multicenter cross-sectional study. BMC Psychiatry.

[58] Cabrera-Aguilar E., Zevallos-Francia M., Morales-García M., Ramírez-Coronel A.A., Morales-García S.B., Sairitupa-Sanchez L.Z., Morales-García W.C. (2023). Resilience and stress as predictors of work engagement: The mediating role of self-efficacy in nurses. Front. Psychiatry.

[59] Khanmohammadi S., Hajibeglo A., Rashidan M., Bekmaz K. (2020). Relationship of resilience with occupational stress among nurse in coronavirus ward of Khatam Al-Anbia Hospital, Gonbad Kovous, 2020. Neuropsychiatr. Neuropsychol..

